# Assessment of Dye-Absorbed Eggshell Membrane Composites as Solid Polymer Electrolyte of Fuel Cells

**DOI:** 10.3390/membranes13010115

**Published:** 2023-01-16

**Authors:** Naoki Tanifuji, Takeshi Shimizu, Kentaro Ida, Kosuke Nishio, Miki Tanaka, Yuta Tsukaguchi, Kentaro Tsubouchi, Akihiro Shimizu, Ei-ichi Hino, Yusuke Date, Kaoru Aoki, Hirofumi Yoshikawa

**Affiliations:** 1 Chemistry and Biochemistry Division, Department of Integrated Engineering, National Institute of Technology, Yonago College, 4448 Hikona-cho, Yonago, Tottori 683-8502, Japan; 2 Department of Material Science, School of Engineering Kwansei Gakuin University, Gakuen 2-1, Sanda 669-1337, Japan

**Keywords:** polymer electrolyte fuel cell, eggshell membrane, dye, absorption ability, hydrophilic groups, dye-absorbed composite membrane, proton conductivity

## Abstract

Recently, polymer electrolytes have been developed for high-performance and eco-friendly fuel cells. Among the candidates, eggshell membrane (ESM) has been promising because of its abundance to assemble various energy devices with low cost and its absorption ability of organic materials. In this work, we investigated fuel cells that included ESM-absorbing xanthene-, triphenylmethane-, and azo-type tar dye, which contained abundant hydrophilic groups, as polymer electrolytes. We found out two points: (1) that the fuel cells that included ESM-absorbing xanthene-type dye generated the highest *I*–*V* performance, and (2) the basic molecular structures of the tar dyes determined the correlation of the maximum power and proton conductivities.

## 1. Introduction

Recently, the fuel cell (FC) has been developed as one of the renewable energy systems without carbon dioxide emissions. In addition, unlike solar power generation and tidal power generation, FCs have also been used widely, such as for home power sources and automobile batteries, regardless of weather or location [1,2]. Among them, polymer electrolyte fuel cells (PEFCs), composed of an electrolyte membrane such as Nafion, current-collector boards, and platinum catalysts, have been most widely used as next-generation power sources because of their higher efficiencies than conventional thermal power generation [3,4,5,6]. A conventional PEFC generates electricity via three steps. First, the oxidation of fuel, such as hydrogen and methanol, added to the anode side, is supported by a Pt catalyst on the electrolyte membrane. Second, protons are transported from an anode to a cathode through the electrolyte membrane polymer Nafion, which has fluorocarbon and sulfonate groups [7], and electron conduction occurs through an electrical conductor. Finally, the reduction of oxygen flowing into a cathode is supported by electrons and protons [4,8,9]. However, Nafion has low thermal stability (the glass transition temperature of Nafion is approximately 100 °C) and requires high humidity, resulting in low generation efficiency. Moreover, gas produced by the combustion of wasted Nafion has a harmful effect on the environment [10,11]. Based on this background, we examined new candidates for the electrolyte membrane to develop both higher-performance and eco-friendlier PEFCs.

**Figure 1:**
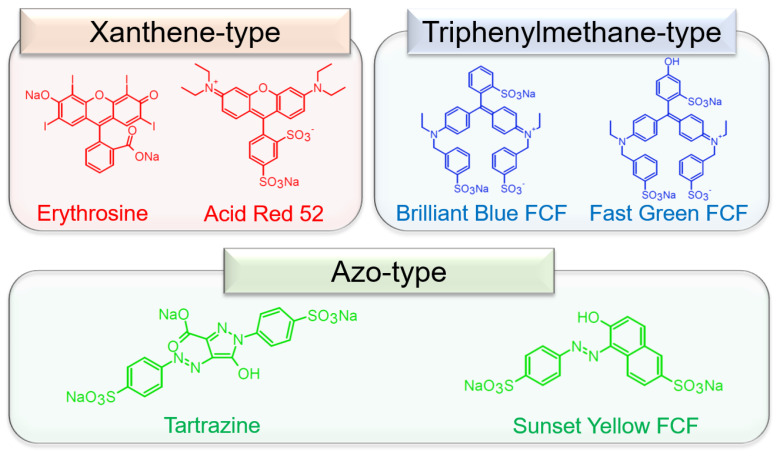
The molecular structures of the tar dyes.

In this research, we report PEFCs, including composites of chicken eggshell membrane (ESM) and tar dyes, xanthene, triphenylene, and azo-type dyes (Figure 1), as the PEFC membrane. Chicken eggs are extensively consumed as they are nutritious, resulting in high costs of mass disposal of eggshells with membranes as industrial waste and garbage [12,13]. Recently, to reduce the costs of mass disposal, ESM has been recycled for various materials, such as the membrane of PEFCs [14] and absorbents [15,16,17,18,19], due to its nanoporous structure composed of protein fibers with abundant amines and amides. In addition, tar dyes contain abundant sulfonic acid and hydroxy groups, suggesting that dye-absorbed ESM (dye–ESM composite) shows higher proton conductivities and enhanced current density compared to pristine ESM [7,20]. In this work, we evaluate the performance of the PEFCs using dye–ESM composites based on a comparison of the structure of tar dyes.

## 2. Materials and Methods

### 2.1. Materials

All materials were used without further purification. Acetic acid (99.7%) was purchased from Kishida Chemical Co. Ltd (Tokyo, Japan). Methanol was purchased from WAKO (Saitama-ken, Japan). Erythrosine, Acid Red 52, Brilliant Blue FCF, Fast Green FCF, tartrazine, and Sunset Yellow FCF as tar dyes were purchased from Tokyo Chemical Industry Co., Ltd (Tokyo, Japan).

### 2.2. Preparation of the Dye–ESM Composites

The dye-ESM composites were obtained by the method in the previous literature with modifications [11]. First, the boiled egg was immersed in 30 wt% acetic acid aqueous solution for two days to remove the eggshell (Figure S1a). The ESM on the equatorial plane was cut into a piece of belt-like sheet (Figure S1b), and the sheet was cut into a square (Figure S1c; area: 3 cm × 3 cm). In addition, to investigate the stability of the pristine ESM, TGA measurements and acid-soaking tests were performed. TGA measurements were carried out with TG/DTA7300 (HITACHI, Hitachi, Japan) from 30 to 500 °C at a heating rate of 5 °C min^−1^ under N_2_. The ESM was soaked in two kinds of strong acids (HCl and H_2_SO_4_). Next, the ESMs were dried in the air at room temperature (approximately 20 °C). Finally, the ESMs were soaked in 1.0 × 10^−2^ M aqueous solutions of each tar dye, and then the dye-ESM composites were dried in the air at room temperature (approximately 20 °C) again.

### 2.3. Scanning Electron Microscopy (SEM) Equipped with Energy Dispersive X-ray Spectroscopy (EDX) Characterization

To observe the morphology of the dye-ESM composites, SEM was performed using a JCM-6610 (JEOL, voltage: 15 kV) under high vacuum. The dye-ESM composites on the carbon tape were sputtered with gold in vacuo for 2 min and three times. The EDX elemental mapping measurements were performed with an SDD (JED-2300; JEOL, Tokyo, Japan) energy dispersive spectrometer equipped with SEM with a magnification of 1600× *g*. The homogeneity of erythrosine on the ESM was investigated because iodine was easily detected.

### 2.4. Proton Conductivity Measurement of Dye–ESM Composites

A Pt sputter coating was applied to both sides of the dye-ESM composites using MSP-1S magnetron-sputtering equipment (VACUUM DEVICE, Tokyo, Japan; Figure S2a) and a Pt target. For coating, 30 μg cm^−2^ of Pt was sputtered on the membrane for 1 min using an acrylic mask with a 1 cm × 1 cm square hole (Figure S2b,c).

We assembled the symmetric cells with the Pt-coated ESMs to calculate the proton conductivities in the ESM thickness direction using the electrochemical impedance spectra (EIS). First, the Pt-coated ESMs were soaked in pure methanol for 1 min. Second, the ESMs were sandwiched with stainless current collectors. The EIS measurements were performed using cells with the ESMs on a Solartron 1296A (AMETEK, Inc., San Diego, CA, USA) at an amplitude of 100 mV in the range of 10 Hz-10 MHz at room temperature (approximately 25 °C). We calculated the proton conductivity *σ* from the following equation:

(1) $$\sigma =\frac{L}{RS}$$

*L*, *R*, and *S* are the membrane thickness, charge transfer resistance, and surface area (1 × 1 cm^2^), respectively. To compare the proton conductivity of the ESM, the same measurement was carried out using Nafion (Sigma-Aldrich, Osaka, Japan; NRE-212, thickness: 0.002 inch) coated with sputtered Pt.

### 2.5. I–V Performance of the Fuel Cells

To evaluate the *I*–*V* performance, fuel cells with the Pt-coated dye-ESMs were assembled by using the parts (Figure S3a). First, as shown in Figure S3b,c, each Pt-coated dye–ESM composite was fixed to current collectors with double-sided conductive tape (width: 1.5 mm; length: 3 cm). Then, the fuel cells were assembled by sandwiching the current collectors with the transparent boards (Figure S3d). The *I*–*V* performance was tested in galvanostatic mode at 25 °C, 60% relative humidity, and a pressure of 1 atm. As the reactant gas, air/air was used in the anode/cathode. The open circuit voltage *V*_oc_ (mV), short-circuit current density *I*_sc_ (µA cm^−2^), and maximum power density *P*_max_ (µW cm^−2^) were measured with a multi-channel recorder at 1 min intervals to collect data for the *I*–*V* curves. To compare the data, the *I*–*V* curves of the PEFC, including Nafion coated with sputtered Pt, were also collected under the same conditions.

## 3. Results and Discussion

**Figure 2:**
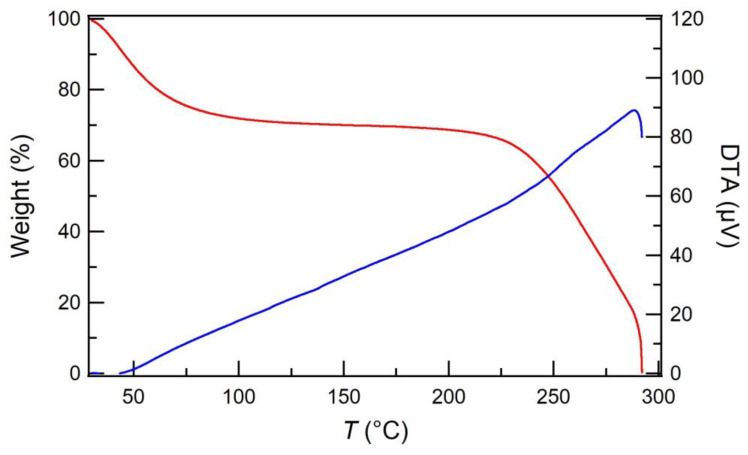
TGA (red) and DTA (blue) curves of the pristine ESM.

The thermal and acid stability of the ESM, which has an effect on the stable PEFC operation, was investigated. The TG curve of the pristine ESM is shown in Figure 2. The initial approximately 30% of mass loss was ascribed to moisture evaporation, and the constant mass area was observed between 75 and 225 °C. The DTA curve shows the only endothermic peak at 288 °C, corresponding to eggshell membrane degradation. In addition, the ESM was stable, even in strong acid (Figure S4). Therefore, the ESM has novel thermal and acid stability, indicating that the ESM serves as the membrane to stabilize the PEFC operation under harsh environments.

**Figure 3:**
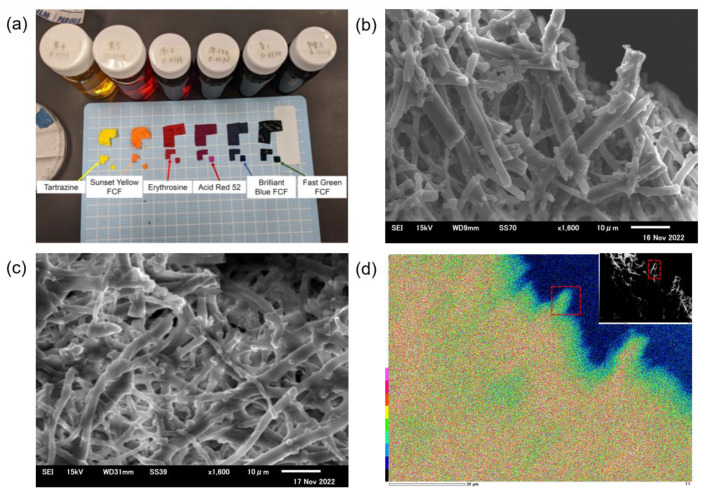
(**a**) The colors of the dye-ESM composites. The SEM images of the erythrosine-ESM composite (**b**), and the pristine ESM (**c**) with a magnification of 1600× *g*. (**d**) The EDX mapping of the erythrosine-ESM composite; inset: the EDX mapping region of the erythrosine-ESM composite with a magnification of 1600× *g*.

To predict proton conduction paths, the distribution of erythrosine was observed. Figure 3a shows the color of each dye-ESM composite. The color of ESM changed evenly into that of each tar dye solution, suggesting that the tar dyes were absorbed in the ESM. Figure 3b show that the interwoven fibers of the erythrosine-ESM composite were similar to those of the pristine ESM (Figure 3c and Figure S5a,b), indicating that the ESM structure was maintained after absorbing tar dyes. Furthermore, EDX mapping (Figure 3d) showed that iodine in erythrosine was detected homogeneously on the ESM. Especially, as shown in the region encircled by the dotted lines, iodine was observed on the ESM fibers. This means that the proton conduction would be promoted by the tar dyes embedded on the ESM fibers.

**Figure 4:**
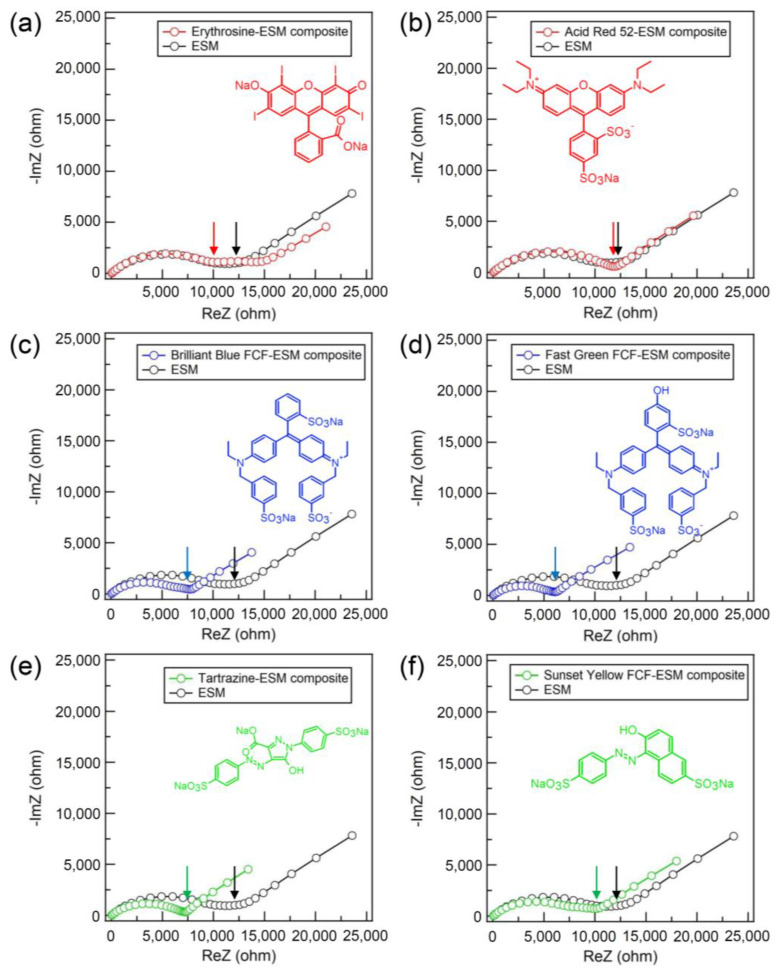
The Nyquist plots of the dye-ESM composites and the pristine ESM. The arrow points the charge transfer resistances *R* of each ESM. Xanthene-type (red): erythrosine (**a**) and Acid Red 52 (**b**); triphenyl-type (blue): Brilliant Blue FCF (**c**) and Fast Green FCF (**d**); azo-type (green): Tartrazine (**e**) and Sunset Yellow FCF (**f**).

**Figure 5:**
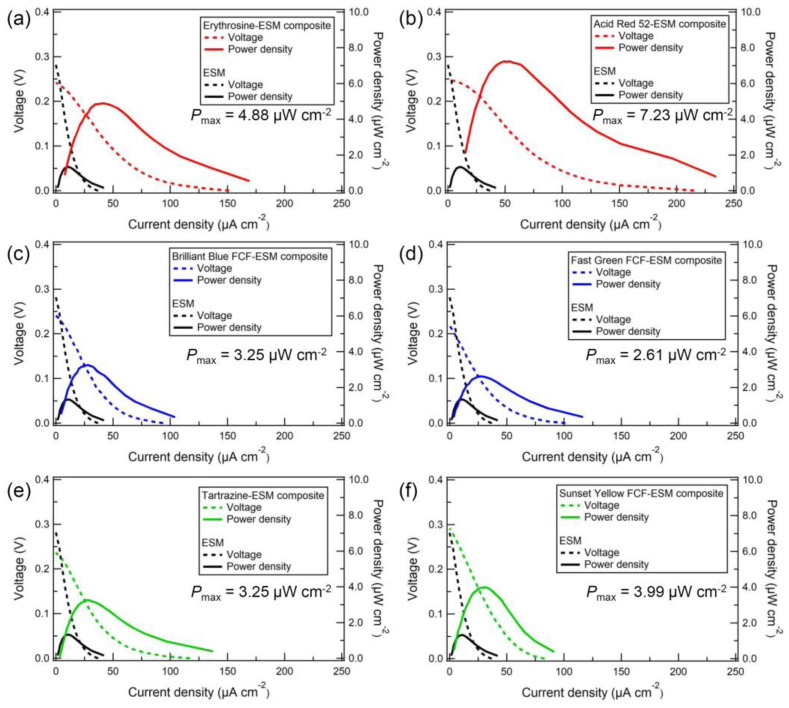
The *I*–*V* curves of dye-ESM composites. Xanthene-type (red): erythrosine (**a**) and Acid Red 52 (**b**); triphenyl-type (blue): Brilliant Blue FCF (**c**) and Fast Green FCF (**d**); azo-type (green): Tartrazine (**e**) and Sunset Yellow FCF (**f**).

**Figure 6:**
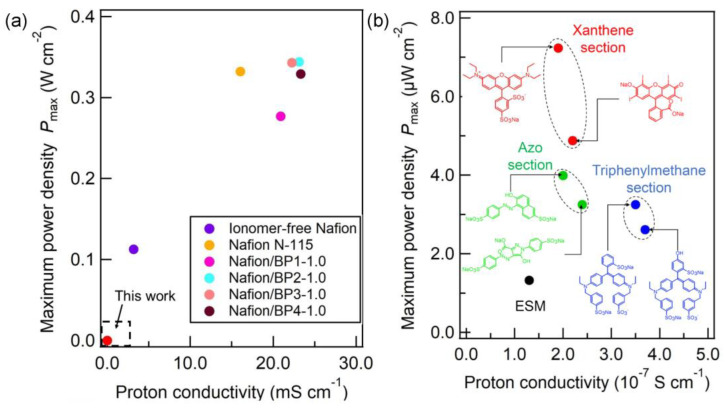
The correlation between the proton conductivities and the maximum power densities of ionomer-free Nafion-based PEMFCs [24,25] (**a**) and dye-ESM composites (**b**).

Figure 4a–f show the Nyquist plots of the dye-ESM composites and the pristine ESM. The charge transfer resistance *R*, corresponding to the right end of the semicircle, was equal to the ionic resistance of each membrane [21]. The tail in the low-frequency region on the Nyquist plots indicated Warburg impedance, which resulted from the diffusion process of proton carriers. The charge transfer resistances of triphenylmethane- and azo-type dye-ESM composites were lower than the charge transfer resistances *R* of the pristine ESM. The triphenylmethane- and azo-type dye-ESM composites had lower charge transfer resistances *R* than did the pristine ESM. The charge transfer resistances *R* of the xanthene-type dye-ESM composites were nearly equal to that of the pristine ESM because the xanthene-type dye-ESM composites were 1.5 thicker than the pristine ESM. The calculated proton conductivities *σ* were higher than that of the pristine ESM (*σ* = 1.3 × 10^−7^ S cm^−1^). In particular, phenylene-type dyes, Brilliant Blue FCF (*σ* = 3.5 × 10^−7^ S cm^−1^) and Fast Green FCF (*σ* = 3.7 × 10^−7^ S cm^−1^) showed the highest proton conductivities of the tar dyes. [22] These indicate that the tar dyes supported the proton conduction in the ESM via their hydrophilic groups, such as hydroxy and sulfonate, in the tar dyes embedded on the ESM fibers. In addition, Figure 5a–f show the *I*–*V* curves of the PEFC using the dye-ESM composites. The xanthene-type dyes exhibited the maximum power densities *P*_max_ (erythrosine: 4.88 µW cm^−2^, Acid Red 52: 7.23 µW cm^−2^) although their proton conductivities were lower than those of triphenylene-type dyes (the maximum power densities *P*_max_ of phenylene-type dyes, Brilliant Blue FCF: 3.25 µW cm^−2^, Fast Green FCF: 2.61 µW cm^−2^). In addition, the *I*–*V* curves of the PEMFC without ionomer (Figure S6) were collected to compare the performance of the PEMFC with the ESM. The Nafion-PEMFC performance shows that without the ionomer, Nafion had low PEMFC performance, equal to that of the ESM. On the other hand, the dye-ESM composite improved the PEFC performance, suggesting that dye might serve as the ionomer. Furthermore, as shown in Figure 6, the basic molecular structure determined the correlation between proton conductivities *σ* and maximum power densities *P*_max_ of the dye-ESM composites. This correlation means that the power density of PEFCs could be greatly improved by the specific organic molecule structure, especially the xanthene structure, although the power density also could be enhanced by the high proton conductivity. This detail is unclear at present, but it is possible however, that the xanthene structure could promote oxygen reduction on the cathode, resulting in increases in the current density and power density [23].

## 4. Conclusions

In conclusion, the tar dyes promoted the proton conduction in ESM caused by the abundant hydrophilic organic groups in tar dyes. In addition, the dye-ESM composites generated higher power densities than did the pristine ESM, and the basic molecular structures of tar dyes determined the correlation between proton conductivities *σ* and maximum powers *P*_max_. In addition, we found two problems: (1) The dye–ESM composites should be improved if they are used as a substitute membrane for Nafion, which has a high proton conductivity of 7.8 × 10^−2^ S cm^−1^ at RH of 80% due to abundant –SO^3−^ groups and water [26,27]. (2) Unfortunately, the PEMFC, including ESM, exhibited lower performances than the previous PEMFCs (Table S1) [28,29,30]. This is because the inactive platinum catalyst might exist due to gaps between the catalyst layer on the ESM and the electrodes produced by the thickness of the double-sided tape. As a solution, conductive carbon paper will be used as a gas diffusion layer to fill the insulating gaps. However, the dye–ESM composite is one of the candidates for the PEFCs under harsh environments, such as low humidity, high temperature, and strong acid conditions. We believe that these findings will contribute to the development of eco-friendly and high-performance PEFCs.

## Data Availability

Not applicable.

## Supplementary Materials

The following supporting information can be downloaded at: https://www.mdpi.com/article/10.3390/membranes13010115/s1, Figure S1: Preparation of the ESM; Figure S2: The Pt-sputter coating method; Figure S3: The assembly of the PEFCs using ESM; Figure S4: The chemical stability of the pristine ESM to strong acids (HCl and H_2_SO_4_). Figure S5: SEM images of the front (a) and back (b) of the pristine ESM. The ESM has the interwoven fiber structure with too tiny pores to observe by using SEM; Figure S6: The *I*–*V* curve of the FC using Nafion; Table S1: Comparison of performances among the ionomer-free PEMFCs.
