# Supplementary material for: Assessment of Dye-Absorbed Eggshell Membrane Composites as Solid Polymer Electrolyte of Fuel Cells
Source: Membranes (Basel). 2023 Jan 16;13(1):115. doi: 10.3390/membranes13010115 (PMC9866477; doi:10.3390/membranes13010115)
Supplement: Supplementary file 1 [file membranes-13-00115-s001.zip › membranes-2013981-supplementary.pdf]

Supplementary Materials

Assessment of Dye-absorbed Eggshell Membrane Composites as Solid Polymer Electrolyte of Fuel Cells

Naoki Tanifuji*, Takeshi Shimizu *, Kentaro Ida, Kosuke Nishio, Miki Tanaka, Yuta Tsukaguchi, Kentaro Tsubouchi, Akihiro Shimizu, Ei-ichi Hino, Yusuke Date, Kaoru Aoki, Hirofumi Yoshikawa*

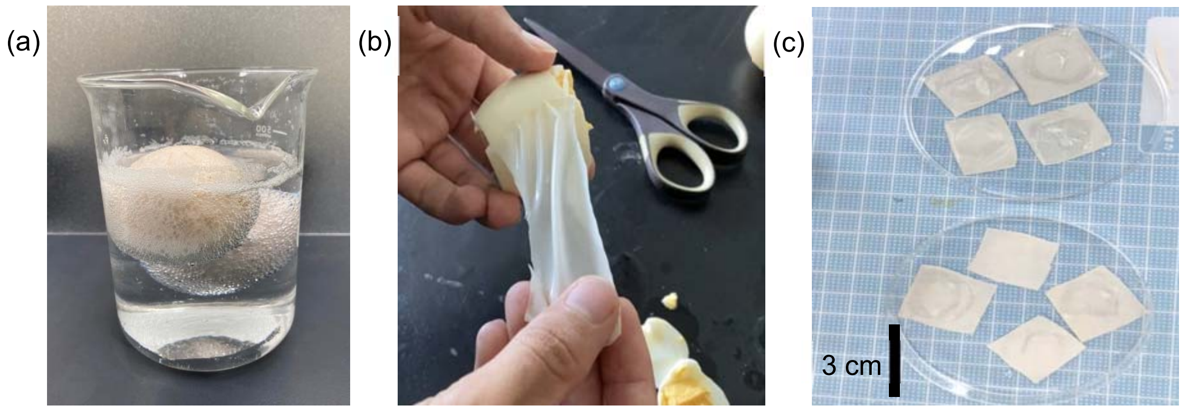

Figure S1. Preparation of the ESM. (a) the boiled egg immersed in 2 M HCl. (b) The belt-like ESM. (c) The square-shaped ESM.

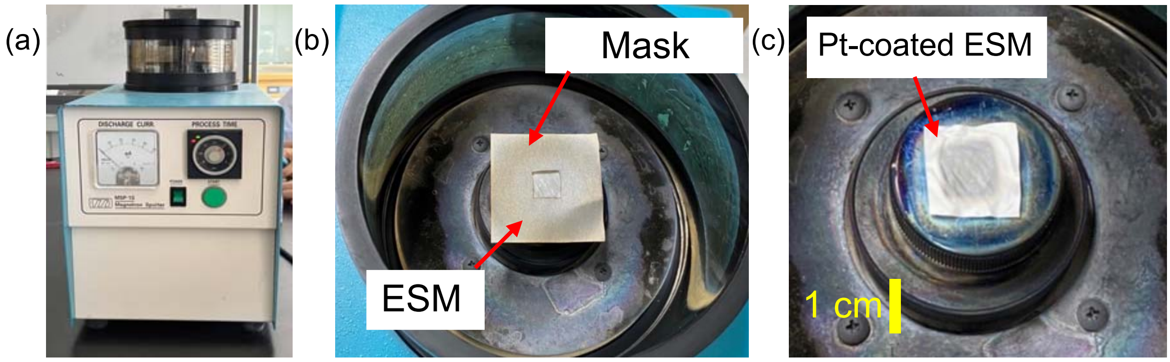

Figure S2. The Pt-sputter coating method. (a) The photograph of the magnetron sputtering equipment, MSP-1S. (b) The ESM covered with the mask to sputter Pt. (c) The Pt-coated ESM.

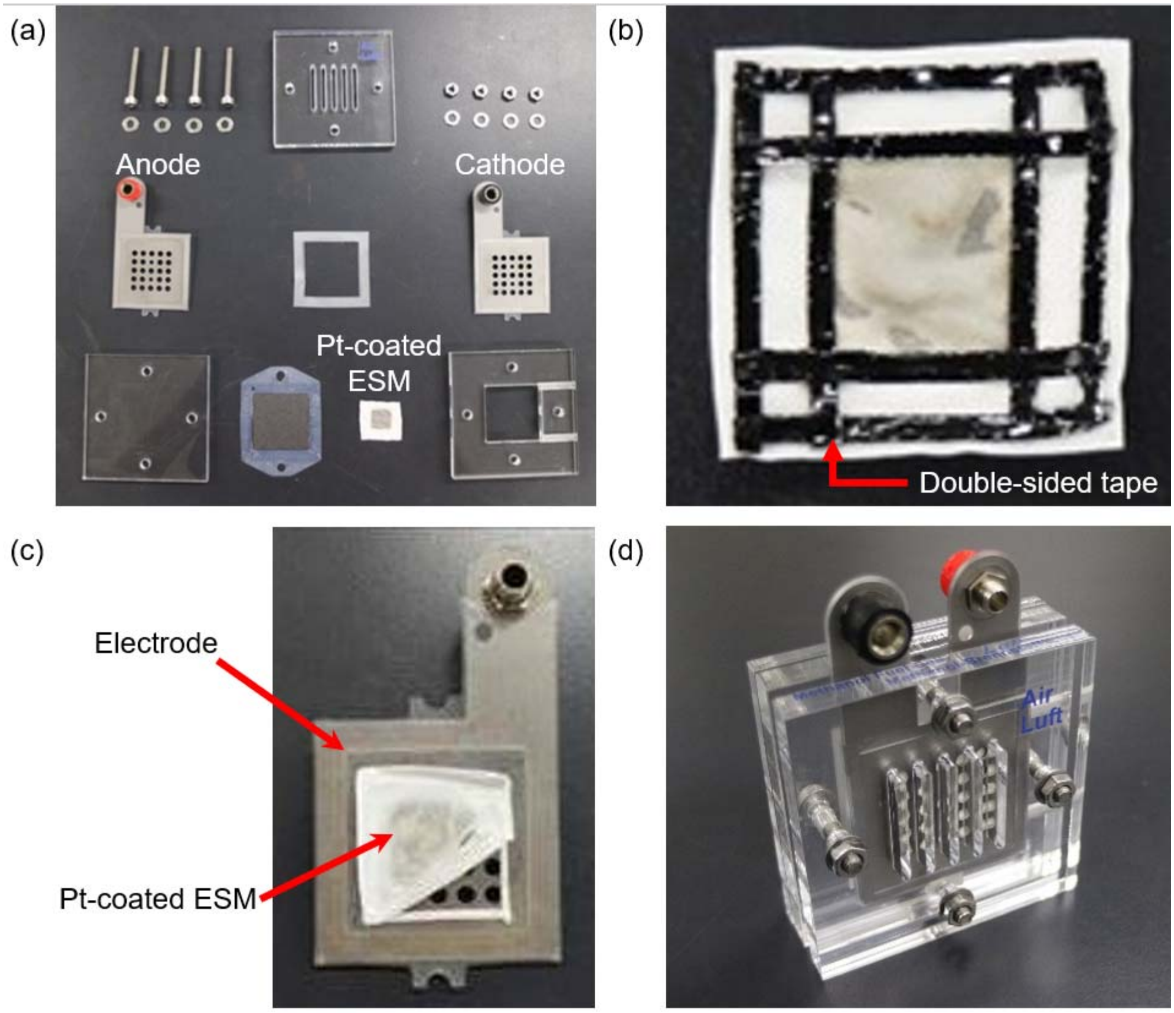

Figure S3. (a) The assembly of the PEFCs using ESM. (a) The components of the PEFC. (b) The Pt-coated ESM attached with the double-sided tapes. (c) Pt-coated ESM attached to the electrode. (d) The assembled PEFC.

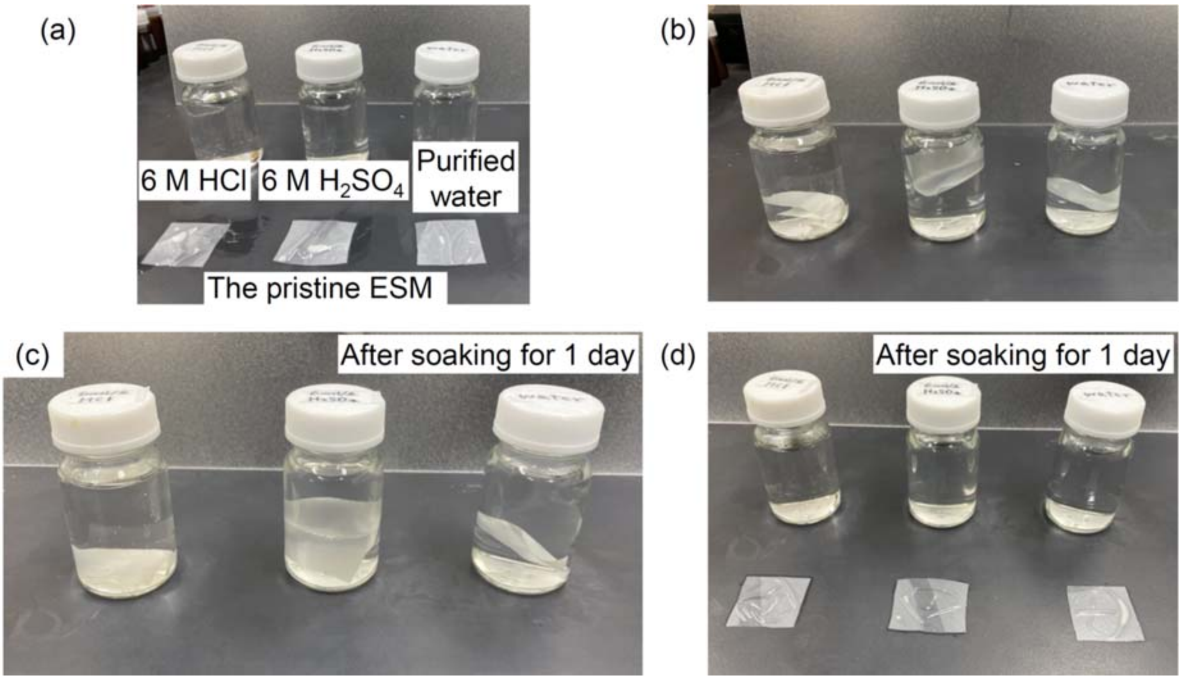

Figure S4. The chemical stability of the pristine ESM to strong acids (HCl and H_2SO_4). (a) Before soaking the pristine ESM in 6 M HCl, 6 M H_2SO_4, and purified water. (b) The pristine ESM soaked in 6 M HCl, 6 M H_2SO_4, and purified water. (a) The pristine ESM soaked in 6 M HCl, 6 M H_2SO_4, and purified water for one day. (d) After soaking the pristine ESM in 6 M HCl, 6 M H_2SO_4, and purified water.

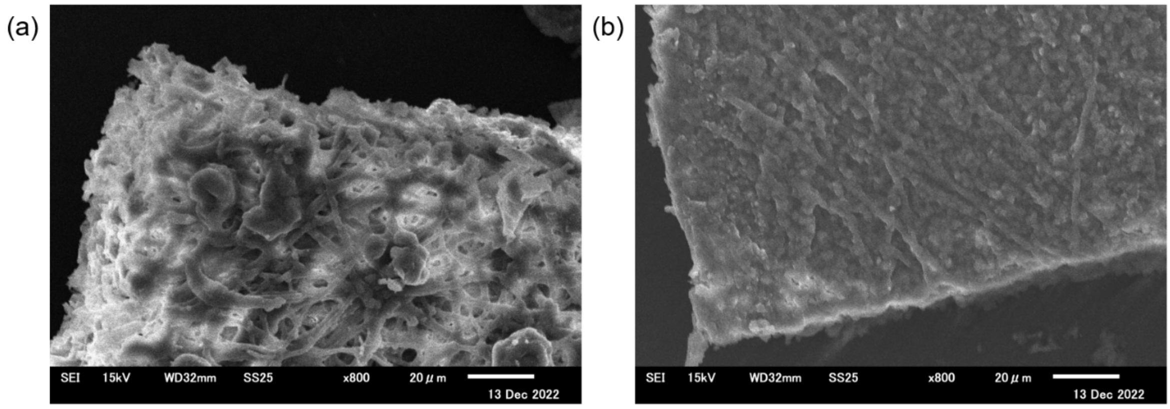

Figure S5. SEM images of the front (a) and back (b) of the pristine ESM. The ESM has the interwoven fiber structure with too tiny pores to observe by using SEM.

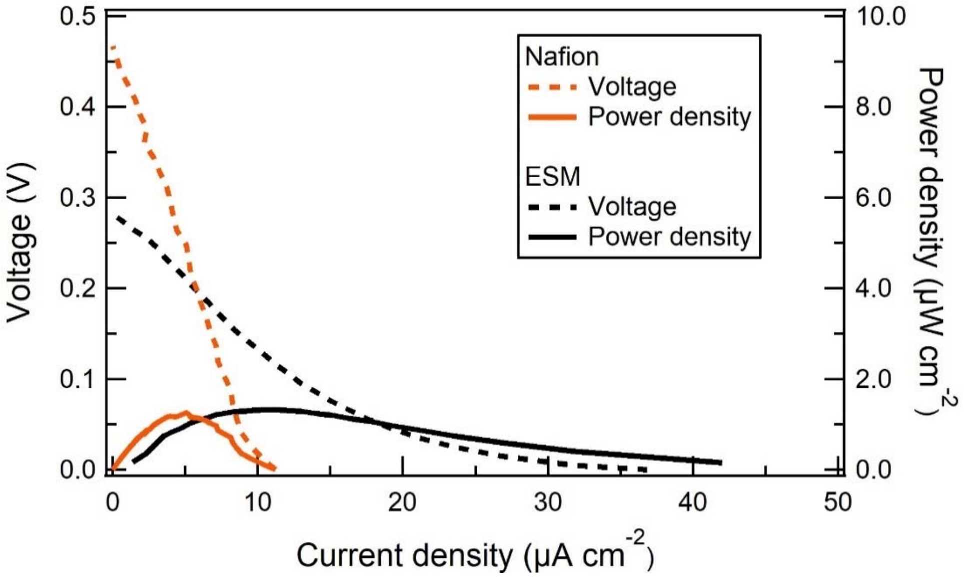

Figure S6. The *I-V* curve of the FC using Nafion.

Table S1. Comparison of performances among the ionomer-free PEMFCs.

**Materials**	**Pt loading [μg cm^{-2}]**	**Maximum power density [W cm^{-2}]**	**Ref**
MPL2A	8.5	1.0-1.2	28
MPL2B	3.4	0.5-0.7	
Sputtered-Pt/C	20	1.24	29
CeO_y/CN_x	1.7	0.54-0.65	30
ESM	30	1.32×10^{-6}	**This work**
Erythrosine-ESM	30	4.88×10^{-6}	**This work**
Acid Red 52-ESM	30	7.23×10^{-6}	**This work**
Brilliant Blue FCF-ESM	30	3.25×10^{-6}	**This work**
Fast Green FCF-ESM	30	2.61×10^{-6}	**This work**
Tartrazine-ESM	30	3.25×10^{-6}	**This work**
Sunset Yellow FCF-ESM	30	3.99×10^{-6}	**This work**

MPL: carbon microporous layer
